# The feasibility of a crossover, randomized controlled trial design to assess the effect of probiotics and prebiotics on health of elite Swiss para-athletes: a study protocol

**DOI:** 10.1186/s40814-022-01048-6

**Published:** 2022-04-27

**Authors:** Marija Glisic, Joelle L. Flueck, Belinda Ruettimann, Anneke Hertig-Godeschalk, Ezra Valido, Alessandro Bertolo, Gerold Stucki, Jivko Stoyanov

**Affiliations:** 1 Swiss Paraplegic Research, Guido A. Zäch Strasse 4, CH-6207 Nottwil, Switzerland; 2 Institute of Social and Preventive Medicine (ISPM), University of Bern, Mittelstrasse 43, CH-3012 Bern, Switzerland; 3 Sports Medicine, Swiss Paraplegic Centre, CH-6207 Nottwil, Switzerland

**Keywords:** Spinal cord injury, Probiotic, Prebiotic, Feasibility trial, Clinical trial design

## Abstract

**Background:**

Spinal cord injury (SCI) may cause an autonomic imbalance in the gastrointestinal tract, leading to deficits in colonic motility, mucosal secretions, vascular tone, and an increase of intestinal barrier permeability. Autonomic denervation and factors such as age, physical activity, antibiotic use and stress may cause intestinal bacterial translocation, decreased microbiota diversity, known as gut dysbiosis and thus increase susceptibility to experiencing gastrointestinal discomfort. Probiotic treatment in individuals with SCI may normalize the gut microbiota and improve overall health. We aim to assess the feasibility of probiotic and prebiotic intervention in athletes with SCI and collect information necessary for sample size calculation of a definite trial on improving health outcomes in para-athletes.

**Methods and analysis:**

Elite Swiss para-athletes (aged> 18 years), being shortlisted for the Paralympic Games 2021 in Tokyo or a member of a national team (*n* = 43), will be invited to participate in this single-center randomized crossover trial. Athletes suffering from chronic inflammatory bowel diseases, those currently taking antibiotics or other medication to alleviate gastro-intestinal complaints will not be eligible to be included in the study. Athletes will be randomized (1:1) to receive for 4 weeks a daily dose of either 3 g of probiotic preparation or 5 g of prebiotic (organic oat bran) supplementation in addition to usual diet, followed by a 4-week washout period or vice versa. The primary outcome is the feasibility of the study, measured by recruitment and dropout rates, feasibility of the measurements, acceptability and adherence to the intervention. Secondary outcomes include gastrointestinal health assessment, diet and training information, handgrip strength, blood diagnostic parameters, and intestinal microbiome characterization. The changes in clinically relevant secondary outcome values will be used to make a power calculation for definite trial.

**Discussion:**

This pilot trial will address two common challenges in SCI research: the difficulty to recruit enough participants for a sufficiently powered study and the ability to collect data within the limits of a realistic budget and time frame. Upon demonstrated feasibility of the intervention and study procedures, the intervention will be evaluated in a definitive controlled trial comprising a larger sample of para-athletes (elite, engaged, or recreationally active) individuals with a SCI.

**Trial registration:**

NCT04659408

## Introduction

Spinal cord injury (SCI) disrupts the autonomic nervous system, impairs its ability to coordinate organ function and leads to SCI–immune depression syndrome and autonomic dysreflexia [1]. The autonomic imbalance in the gastrointestinal (GI) tract can affect colonic motility, mucosal secretions and intestinal barrier permeability [2, 3]. Besides causing GI discomfort, these changes cause variations in the composition of the gut microbiota and create a state of dysbiosis in which the balance between beneficial bacteria and pathogenic bacteria is skewed towards favoring pathogenic species [1]. Furthermore, prolonged and repeated antibiotic use, malnutrition, physical inactivity and stress may exacerbate the effects of autonomic dysreflexia on the gut microbiome [4]. In addition, gut microbiota generates biologically active metabolites that may affect host's phenotypes relevant to cardiovascular disease (CVD) development, ranging from inflammation, obesity, insulin resistance, tissue cholesterol balance and thrombosis risks [5, 6].

Diet may be the key modifiable factor influencing microbiome and metabolic and gastrointestinal health [7]. Although SCI-specific dietary guidelines recommend caloric restriction and a heart-healthy nutrition plan focusing on fruits, vegetables, whole grains, low-fat dairy, poultry, fish and legumes [8, 9], the chronic SCI population does not adhere to dietary recommendations [10–14]. A recent systematic literature review explored nutritional status in chronic SCI [15]. The findings indicated greater energy intake relative to energy needs in those with chronic SCI, and an imbalance in fiber intake and macronutrients (excessive protein and carbohydrate intake) and micronutrients (deficiency of vitamins A, B5, B7, B9, D, E and potassium and calcium) compared to the U.S. Department of Agriculture (USDA) guidelines [15]. In contrast to the general SCI population, competitive athletes are in constant need for optimal nutritional approaches to improve training stimulus and maximize their performance [16]. This means, that athletes often follow a well-balanced diet, designed to ingest a sufficient amount of protein, carbohydrates, fatty acids, vitamins and minerals to cover their macro- and micronutrient requirements. Exercise, in addition, improves GI function [17] and protects against GI disease [18]. Thus, a microbiome of professional athletes seems to have a better profile compared to the microbiome of sedentary individuals [19].

However, in the days before a competition, athletes might adhere to a protein-rich, low fiber diet, which might change bowel motility and gut microbiota [20]. Furthermore, travelling to and change of diet during a competition might further negatively influence the GI system. In a worst-case scenario, significant exercise-constraining GI problems such as cramps and diarrhea may occur during the competition [21]. Probiotics containing various beneficial microbial species, and prebiotics can support and enhance healthy microflora. Both are linked with anti-inflammatory, hypoglycemic, insulinotropic, and antioxidative properties; thus, they may partially compensate pathophysiologic effects of gut dysbiosis and improve short and long-term health in this vulnerable population [22, 23]. Therefore, probiotic supplementation is recommended in order to prevent GI problems during competition or while travelling [24].

Therefore, we hypothesize that probiotic intervention in athletes with SCI may elicit positive changes in GI symptoms, gut microbiome composition and overall health, and potentially lead to improved sport performance. Considering the scarcity of evidence, vulnerability of the target population and peculiarity of dietary and exercise regimes in para-athletes, it is necessary to conduct a pilot study to examine the feasibility of pro- and prebiotic intervention in this population and collect information necessary for sample size calculation of a subsequent trial. In this study, we describe the protocol of a pilot study that assesses the feasibility of a crossover, randomized controlled trial design to assess the effect of probiotics and prebiotics on health of elite Swiss wheelchair para-athletes.

## Methods

### Study design, setting, and sample size

**Fig. 1:**
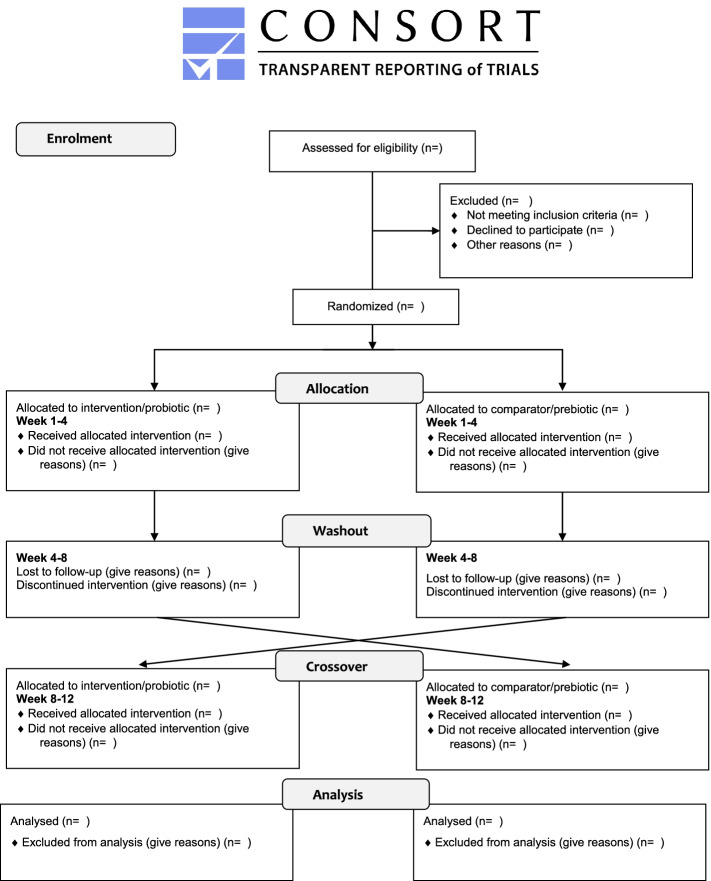
The participant flow diagram

**Fig. 2:**
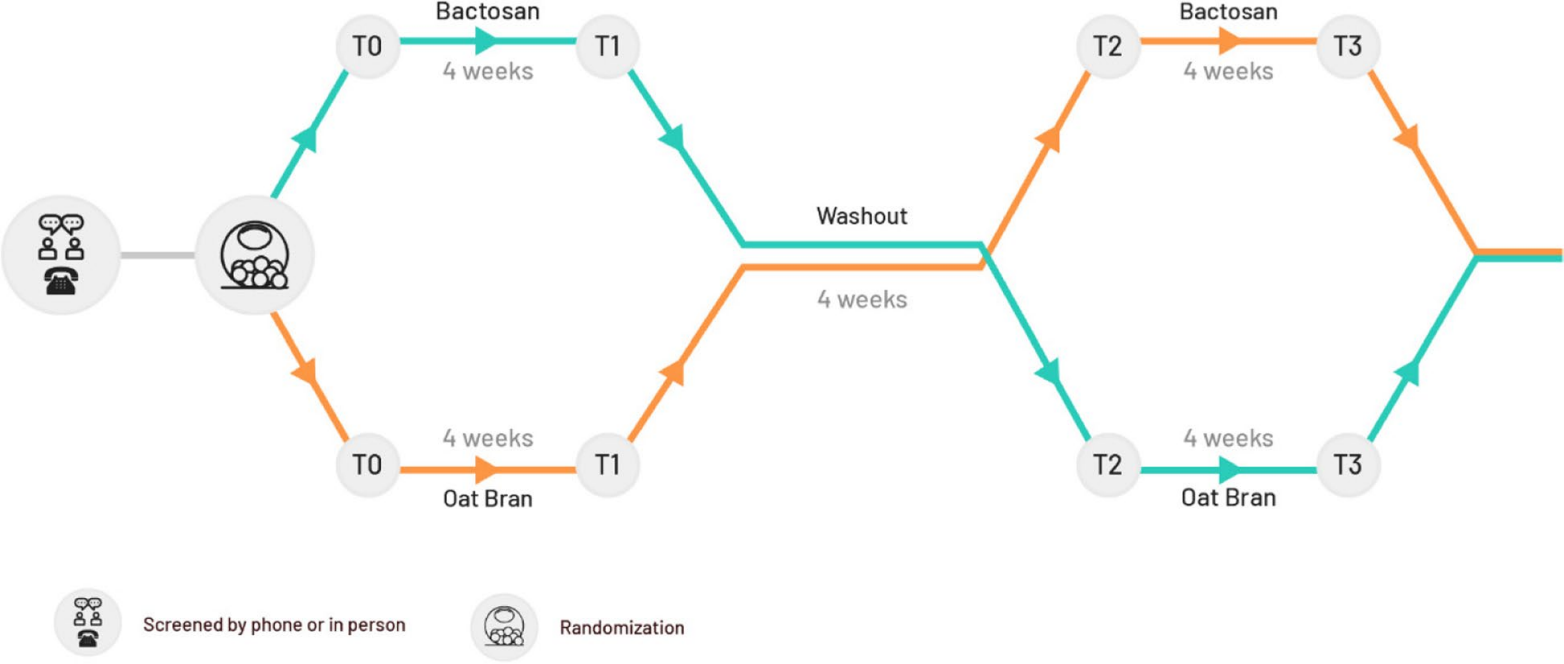
Summary of study design. Participants are randomized to either probiotic (Bactosan pro-FOS) or prebiotic (oat bran), both taken once a day for 4 weeks (T1), followed by a washout period of 4 weeks (T2) and cross-over to the other intervention for another 4 weeks (T3)

This is a single-center randomized controlled crossover trial adhering to the CONSORT guidelines (Fig. 1) [25] with a total study duration of 12 weeks. A cross-over design offers two advantages over a parallel-group RCT: (1) the influence of confounding covariates are reduced because each participant serves as their own control; and (2) statistical power is higher and required sample size to detect meaningful effects is lower [26]. The study takes place at the Sports Medicine Department within the Swiss Paraplegic Center, the largest SCI-specialized hospital in Switzerland. Each year around 100 wheelchair athletes visit the Sports Medicine Department to perform medical and performance check-ups, thus making a pool of potential study participants. The intervention involves of a 4-week intervention consisting of a daily intake of a multistrain probiotic preparation, or oat bran as prebiotic comparator, followed by a 4-week washout period, and vice versa for four additional weeks (Fig. 2). The method for allocating to intervention (probiotic) or comparator (prebiotic) will be a 1:1 randomization with blocks of two and four executed by a Good Clinical Practice compliant data management system (secuTrial®, interActive Systems, Berlin).

Sample size estimates for the pilot study were done according to published recommendations for minimizing a pilot trial sample size [27]. Here, the use of the stepped rule is recommended in case the standardized effect size to be used in the main trial is unknown. Thus, for a main trial designed with 80% power and two-sided 5% significance, recommended pilot trial sample sizes per treatment arm of 50, 20, 10, and 10 for standardized effect sizes that are extra small (≤ 0.1), small (0.2), medium (0.5), or large (0.8), respectively [27]. We expect to observe a small effect size in the current pilot study and, therefore, we aim to recruit 20 study participants.

### Study participants

Swiss male and female para-athletes above the age of 18 and who are shortlisted for the Paralympic Games 2021 in Tokyo (*n* = 28) or member of a national team (*n* = 15) will be invited to participate. The larger subsequent trial will be recruiting participants among all eligible athletes regularly visiting the Sports Medicine Department and participating in wheelchair sports on a national level (*N* = 100). Exclusion criteria are the presence of chronic inflammatory bowel diseases (ulcerative colitis, Crohn’s disease), taking antibiotics or immunosuppressive drugs, or pregnancy at the beginning of the study. Potential participants will be approached, informed about the study, and screened by phone or in person. After given at least 24 h to consider participation, informed consent will be obtained from all participants. Participants will be redrawn in the following cases: retraction of informed consent, occurrence of a serious adverse advent, a serious sports injury or when any other eligibility criterium is compromised.

### Intervention

#### Probiotic intervention

The probiotic period involves a 4-week supplementation with the commercially available freeze-dried multispecies probiotic preparation Bactosan pro FOS (Mepha, Basel, Switzerland) in addition to usual diet. Following the product guide, the content of one sachet (3 g) will be mixed with 50-100 ml water and taken once daily on an empty stomach before a meal of choice (preferably breakfast). Bactosan pro FOS contains the following bacterial strains: *Bifidobacterium lactis W51, Bifidobacterium lactis W52, Enterococcus faecium W54, Lactobacillus acidophilus W22*, *Lactobacillus paracasei W20*, *Lactobacillus plantarum W21*, *Lactobacillus salivarius W24*, and *Lactococcus lactis W19,* with a total viable cell count of 1 × 10^9^ cfu/g, and therefore 3 × 10^9^ cfu/daily dose. To ensure osmotic stability, survival and biological activity in the GI tract, the following ingredients have been added to the bacterial strains: inulin, fructo-oligosaccharides, amylases, maltodextrin, maize starch and a mineral mix (combination of potassium chloride, magnesium sulphate, and manganese sulphate).

#### Prebiotic comparator

The control period involves a 4-week supplementation with oat bran (Naturaplan, Coop, Switzerland). The participants will consume daily 5 g (one teaspoon) of the oat bran together with a meal of choice in addition to usual diet. The oat bran can be added to the usual breakfast cereal, yoghurt, pancakes, juice, milk, or in an omelet. Based on the findings from a recent systematic review, 40 to 100 g/day of oat bran was shown to increase fecal bacterial mass and short-chain fatty acids in humans [28]. Therefore, we do not expect that the amount of 5 g per day of oat bran cause significant physiological changes in individuals with SCI.

#### Washout period

A 4-week wash-out period was chosen based on the review by Roberfroid [29]. Interventions using inulin-type fructans lead to increases in *Bifidobacteria* which become significant and maximal in less than a week, remain as long as the intake of the prebiotic continues and progressively disappear when the intake stops (within 1–2 weeks) [29]. Although a wash-out period of 2 weeks has been suggested to be sufficient, we extended it to 4 weeks to decrease the possibility of carryover effect.

### Primary outcome measures

#### Feasibility outcomes

Feasibility outcomes were established to explore the methodological, procedural, and clinical uncertainties of the study. These outcomes comprise the recruitment rate, appraisal of the eligibility criteria, completeness of data collection, adherence to the intended supplementation intervention, acceptability of the intervention, participant retention, resources needed to complete the study, safety analyses as well as power analysis of sample size for larger randomized controlled trial (RCT) (the difference between intervention and control/comparator group, and standard deviation). Furthermore, the acceptability of the study among participants will be evaluated post-trial by a combination of open and closed questions regarding the satisfaction with for example the intervention and time burden. All feasibility outcomes are shown in Table 1 alongside the associated progression criteria. The progression criteria are set to facilitate the interpretation of the results and to help us deciding on whether, as well as how, to proceed to a definite trial after the feasibility study. In addition, the results of this pilot study will provide information necessary for the sample size calculation of the future (full-scale) trial.

**Table 1:** Overview of feasibility outcomes and progression criteria

Feasibility criteria	Assessment	Progression criteria to a subsequent trial^**a**^
Recruitment and eligibility	Number of eligible athletes	N/A
	Percentage assessed for eligibility; fulfilling inclusion criteria, recruited and included (of total number identified)	> 25% agreed to participate of total eligible population
	Reasons for ineligibility and non-participation	Information to be descriptively summarized. If necessary inclusion/exclusion criteria are to be reconsidered.
Data collection	Percentage of completed assessments/questionnaires	> 75% answering all questions at all assessments
	Numbers of missing items (e.g., laboratory measurements), reasons for missing data collection	< 20% missing information for primary outcomes [30]
Adherence to intervention	A rate of adherence to protocol for both intervention and control interventions	> 80% of participants at baseline
Retention	The number of randomized participants retained/who managed to complete the study protocol Collect the reasons for premature study termination	> 80% of participants at baseline
Feasibility of procedures and experimental setting	Number of: 1. Fasting blood samples collected 2. Correctly taken stool samples by study participants 3. Successfully analysed microbiome composition	> 75% of analytic procedures successfully completed
Acceptability of intervention	Evaluated among participants post-trial: 1. Rating (from 0 to 10) of study procedures (intervention, measurements, time burden, personal value) 2. Willingness to participate again 3. Open feedback	Having an average of at least 5 points for each rating > 50% willing to participate again
Resources needed to complete the study and the intervention	Length of time required for: 1. Study personnel to collect the data the monthly visits 2. Participants to provide data at the monthly visits	< 60 min to complete questionnaires and physical examinations during monthly study visits
Safety analyses	Number of serious adverse events	No serious adverse events related to the study intervention or other procedures.

^a^If one or more criteria are not met revisions should be considered before proceeding to a definite trial

### Secondary outcome measures

#### Sociodemographic characteristics, clinical variables, and gut microbiome composition

Several demographic characteristics will be collected during the baseline visit (T0, Fig. 2) at the study center, including age, sex, SCI (e.g., lesion level and completeness) and highest educational qualification. Body composition will be determined at the baseline visit using a dual X-ray absorptiometry scan (DXA, enCORE v17 Software, Lunar iDXA Serie).

The following data will be collected at each monthly study visit (T0, T1, T2, and T3) at the study center (Sports Medicine Department within the Swiss Paraplegic Center). Secondary health conditions (e.g. urinary and respiratory tract infections) and self-reported medication use. The presence of GI symptoms in the previous 3 weeks will be assessed using the German version of the questionnaire of Eypasch [31], which evaluates the frequency of 36 GI symptoms on a scale from 0 to 4 (0 = never, 4 = all the time). A detailed 3-day food diary based on self-reported intake as well as a 3-day training record for the 3 days prior to the study visit will be completed. The leisure time physical activity questionnaire for individuals with a SCI (LTPA-Q SCI) will be used to assess activity intensity and duration during the last 7 days prior to the study visit [32]. Questionnaires and diaries will be recorded on paper. As a proxy of strength, the average of three maximum handgrip measurements of both hands will be assessed using the Jamar dynamometer (Jamar Hydraulic Hand Dynamometer, Jamar, Bolingbrook, USA). Blood samples will be taken to analyze key markers (e.g., hemoglobin, ferritin, vitamin D) as well as inflammatory markers. A stool sample will be taken at home by the participants within 3 days prior to the study visit using a commercially available kit (OMNIgene®•GUT, DNA Genotek, Ottawa, Canada). The participants bring the stool sample to the study visit and the sample will be used to analyze the microbiome. The Oslo Sports Trauma Research Centre (ORSTC) Questionnaire [33] evaluating illness and injury as well as loss of training days will be filled out weekly by the participants during the entire study and returned to study staff by email or in paper during the study visits. The ORSTC questionnaire plus, when applicable, a reminder to take the supplements, will be emailed to the participants once per week. Despite a daily supplement intake, we have decided to only remind the participants once per week as to not interfere with them too much.

### Data analyses

Descriptive analyses will assess the feasibility of the intervention and study procedures. Progression criteria listed in Table 1 will be used to determine whether revisions should be considered before proceeding to a definite trial.

An adapted CONSORT diagram for pilot and feasibility studies, Fig. 1, will be used to illustrate the participant flow [34]. Reasons for ineligibility, ambiguities regarding eligibility criteria, and reasons for non-participation and dropouts will be reported at each stage. Follow-up rates and numbers of missing items relating to outcome measures will be calculated and expressed in percentages. To our knowledge there are no clinical trials which investigate the differences in outcomes of interest (e.g., body composition parameters, fasting glucose, high sensitivity C reactive protein, total cholesterol) among the two groups (pre- and probiotic) in athletes with a SCI. Therefore, we aim to assess these parameters in the current pilot trial. In particular, we aim to measure within-participants standard deviation and mean differences between the two groups for the main secondary outcomes. We will use the formula suggested by Chow et al. [35] to calculate the sample size of a definite cross-over trial.

### Quality assurance and safety provisions

Participants will be encouraged to maintain a habitual diet and training routine such that potential health modifications can only be attributed to the intervention of probiotics/prebiotics. All measurements will be conducted by qualified and designated personnel and coded data will be entered in a secure database (secuTrial®). To improve compliance, participants will be reminded weekly to take the supplements and are requested to return empty packages to the study center after the intervention periods. The number of missed supplements will be assessed. During the weekly contact, participants are also asked about potential side effects and tolerance of the supplement. Adverse effects or health risks are not expected as probiotic and prebiotic ingestion appears to be safe and without any known major side effects [24]. Serious adverse events will be documented and reported to the local authorities for the entire study duration, encompassing the signing of the informed consent until completion of the last study procedure including a safety follow-up period. A risked-based approach will be applied for the study monitoring, due to the low risks involved in this study [36].

## Ethics and dissemination

### Ethics

The study is approved by the Ethics Committee for Northwest and Central Switzerland (EKNZ, ID: 2020-02337) and has been registered at ClinicalTrials.gov (NCT04659408). The study will be conducted in accordance with the Helsinki Declaration, ensuring the welfare and rights of all participants. All investigators and study personnel involved will respect the participant’s right to confidentiality, and adhere to the Swiss Federal Act on Data Protection (FADP 235.1).

### Dissemination policy

Results will be published in peer-reviewed journals, presented at scientific conferences, and circulated in a newsletter for individuals with a SCI. Individual study results will be shared with the participants after the study end.

## Discussion

Given the limited number of intervention studies conducted within the SCI population, evaluating the feasibility of the intervention and study procedures are of importance for an informed decision on planning of a consecutive RCT. The significance of the pilot trial is to address competently two of the common challenges in the SCI research: the difficulty to recruit enough participants for a good quality full RCT and the ability to measure different variables within the limits of a realistic budget and time-frame. One of the most important skills, which a new team will develop during this pilot study, is to train the communication and daily operations of working together in a clinical trial including athletes with SCI, medical personal and researchers. For a long-term sustainable collaboration, we should take care for the development of our complementary expertise, plan sufficient further funding, set adequate incentives to the participants and desire to support our athletes to achieve their best performance.

Beyond the purposes of feasibility study will be to answer the research questions is of scientific and healthcare significance and the underlying importance of the expected full RCT findings. For example, gut microbiome is influenced by plethora of exogenous and intrinsic host factors. The SCI-induced changes in the “healthy” gut microbiome, changes in the lifestyle and diet or infections and use of antimicrobial substances may contribute to GI diseases, diabetes, obesity, CVD and chronic inflammation. The capacity to access and remodel the composition and function of the microbiota makes it an attractive target for establishing a link between certain patterns of gut microbiota, the sport physiology and the cardio-metabolic and GI status of the athletes. Pre- and/or probiotic intervention might prove to be a good strategy to counteract certain aspects of gut dysbiosis and to improve the short- and long-term health and sport performance in athletes with SCI. The results of this trial may pave the opportunity for establishment of potential biomarkers, and new tools for prevention and treatment in personalized healthcare strategies. Should probiotic intervention and study procedures be demonstrated to be feasible, the intervention will be evaluated in a definitive controlled trial comprising a larger sample of para-athletes. The subsequent study population can be extended to recreationally active individuals with a SCI and sedentary persons with a SCI who wish to include functional nutrition in their daily routines to improve health and GI functioning.

Finally, the Swiss para-athletes will benefit from an optimal support during the last few months before the Paralympic Games, the major competition happening every 4 years. SCI athletes with neurogenic bowel who will participate in the pilot trial are expected to have a possible benefit, which has been demonstrated for irritable bowel syndrome in the general population [37]. Not only their gut will be prepared to the journey to Japan (e.g., jetlag, hygienic standards, eating habits), but also injury and illness will be monitored weekly. This has been shown to reduce the risk of a major illness or injury due to a fast treatment process based on the close monitoring. Thus, this project will be of major interest concerning research involving the microbiome in SCI, but also athletes and medical staff will benefit in terms of an optimal preparation leading into the Paralympic Games as well as other future competitions.

## Data Availability

In this protocol paper, no study results were reported.
